# Novel Formulation of Fusidic Acid Incorporated into a Myrrh-Oil-Based Nanoemulgel for the Enhancement of Skin Bacterial Infection Treatment

**DOI:** 10.3390/gels8040245

**Published:** 2022-04-15

**Authors:** Mervt M. Almostafa, Heba S. Elsewedy, Tamer M. Shehata, Wafaa E. Soliman

**Affiliations:** 1 Department of Chemistry, College of Science, King Faisal University, Alhofuf 31982, Saudi Arabia; 2 Department of Pharmaceutical Sciences, College of Clinical Pharmacy, King Faisal University, Alhofuf 36362, Saudi Arabia; helsewedy@kfu.edu.sa (H.S.E.); tshehata@kfu.edu.sa (T.M.S.); 3 Department of Pharmaceutics, College of Pharmacy, Zagazig University, Zagazig 44519, Egypt; 4 Department of Biomedical Sciences, College of Clinical Pharmacy, King Faisal University, Alhofuf 36362, Saudi Arabia; weahmed@kfu.edu.sa; 5 Department of Microbiology and Immunology, Faculty of Pharmacy, Delta University for Science and Technology, Gamasa, Mansoura 11152, Egypt

**Keywords:** fusidic acid, myrrh essential oil, nanoemulgel, optimization, antibacterial

## Abstract

Fusidic acid (FA) is renowned as an effective bacteriostatic agent obtained from the fungus Fusidium coccineum, used for treating various eye and skin disorders. The objective of the present study was to develop, characterize, and evaluate the antibacterial activity of a novel FA nanoemulgel for topical skin application. Primarily, various fusidic acid nanoemulsion formulations were fabricated using different concentrations of myrrh essential oil, Tween 80 as a surfactant, and Transcutol^®^ P as a co-surfactant. A Box–Behnken design was employed to select the optimized FA nanoemulsion formulation, based on the evaluated particle size and % of in vitro release as dependent variables. The optimized formula was incorporated within a hydrogel to obtain an FA nanoemulgel (FA-NEG) preparation. The formulated FA-NEG was evaluated for its visual appearance, pH, viscosity, and spreadability, compared to its corresponding prepared fusidic acid gel. In vitro release, kinetic study, and ex vivo drug permeation were implemented, followed by formulation stability testing. The FA-NEG exhibited a smooth and homogeneous appearance, pH value (6.61), viscosity (25,265 cP), and spreadability (33.6 mm), which were all good characteristics for appropriate topical application. A total of 59.3% of FA was released from the FA-NEG after 3 h. The ex vivo skin permeability of the FA-NEG was significantly enhanced by 3.10 ± 0.13-fold, showing SSTF of 111.2 ± 4.5 µg/cm^2^·h when compared to other formulations under investigation (*p* < 0.05). No irritation was observed upon applying the FA-NEG to animal skin. Eventually, it was revealed that the FA-NEG displayed improved antibacterial activity against a wide variety of bacteria when compared to its corresponding FA gel and marketed cream, indicating the prospective antibacterial effect of myrrh essential oil. In conclusion, the recommended formulation offers a promising antibacterial approach for skin infections.

## 1. Introduction

Skin is the largest organ in the human body, regarded as a line of defense against different influencers, including light, heat, external pathogens, and infections [1]. It is considered to be a pathway for delivering various medications intended for treating skin disorders and infections. Delivering drugs through the skin is known as transdermal drug delivery, which has recently attracted great interest owing to its effectiveness and convenience. Transdermal drug delivery systems exhibit many advantages over other conventional dosage forms, since the drug can be localized on the affected area, avoiding first pass metabolism and decreasing the systemic drawbacks [2]. Various formulations can be applied topically, such as ointments, creams, lotions, and gels; however, other innovative formulations have been developed in recent times in order to provide high drug loading capacity and improve drug permeability. Such contemporary formulations were developed by applying advanced techniques known as nanotechnology.

Nanotechnology is a new approach for studying, designing, and developing materials at nanoscale. The developed nanomaterials can reach the targeted site in a controlled manner, achieving the greatest therapeutic effect while lowering the drawbacks [3]. Such nanomaterials for use as nanocarriers have been developed for encapsulating various medications for use in various routes of administration, including the transdermal route [4]. Nanoemulsion (NE) is a kind of nanocarrier that comprises a thermodynamically stable system formed by mixing two immiscible liquids, using a surfactant and a co-surfactant [5]. They are manufactured to ensure proper delivery of the pharmaceutical agents, improving the solubility and, consequently, the bioavailability of poorly water-soluble drugs [6]. Nevertheless, their topical application to the skin is difficult owing to its low viscosity. For that reason, and to improve the rheological behavior of the NE, it should be integrated into a hydrogel base, providing a novel formulation known as a nanoemulgel (NEG).

NEGs are emerging transdermal drug delivery systems, composed mainly of NE gelled using a gelling agent. They have the benefits of incorporating both hydrophilic and hydrophobic drugs [7]. Their components provide a drug reservoir that facilitates the drug’s release, increases its absorption, and enhances the penetration into the skin [8]. They have various advantages, since they are thixotropic due to the presence of the gelling agent, which provides the formulation with definite capability to be applied topically [9]. Moreover, nanoemulgels are greaseless, non-toxic, and non-irritant, and offer better spreadability than other conventional topical formulations, which could make them a better candidate for transdermal drug delivery [10]. Nanoemulgels can be utilized as a potential vehicle applied widely for the delivery of analgesics and anti-inflammatory drugs [11], anticancer drugs [12], wound-healing agents [13], antifungals [14], and antibacterials [15].

Antibacterials are well-known as antibiotics or antimicrobial agents that are targeted toward bacteria to overwhelm and suppress their reproduction. Fusidic acid (FA) is one of the most broadly used steroid-type antibacterial agents, and is derived from the fungus *Fusidium coccineum* [16]. It is commonly used for treating primary and secondary skin diseases [17], as well as eye infections [18]. It exhibits effectiveness against Gram-positive bacteria—mostly *Staphylococcus aureus* and *Staphylococcus epidermidis* [19]. Although FA is available in various conventional dosage forms—namely, eye drops, creams, and ointments—its poor water solubility represents a great obstacle in its formulation. On the other hand, essential oils are natural substances extracted from plants, and have been extensively incorporated into various formulations owing to their safety and efficacy [20]. Myrrh oil is an essential oil that has been established to show various therapeutic actions and biological activities [21]. It is used in treating several diseases, since it exhibits anticancer, antioxidant, antifungal, and antibacterial activity [22,23]. In the same vein, incorporating FA into a nanoemulgel formulation prepared with myrrh oil could feasibly modify the influence of the drug and improve its action for transdermal delivery.

Quality by design (QbD) is a novel systematic technique established in order to optimize the developed formulations. Box–Behnken design (BBD) is one of the most commonly applied of these approaches, as it investigates three levels for each factor while requiring fewer of trials [24]. It examines the effects of definite independent variables on the observed dependent responses, so as to obtain the best selected formula with extreme characteristics [12].

Based on these insights, the goal of the present study was decided. FA was incorporated into an NE formulation fabricated with myrrh essential oil, which was later mixed with a pre-prepared hydrogel base. A 33 full factorial design was constructed, followed by optimizing the best nanoemulgel formulation to be explored for its antibacterial behavior.

## 2. Results and Discussion

### 2.1. Experimental Design with BBD

#### 2.1.1. Fitting the Model

In line with the data shown in Table 1, 15 experimental formulae were developed using BBD software. The formulations were generated using three independent factors that exhibited their impact on two dependent responses.

#### 2.1.2. Analysis of the Data

In the present investigation, the best fit model for the responses R_1_ and R_2_ was the quadratic model, compared to other models in the design. Regarding *p*-values, it was necessary that they be less than 0.05 in most of the model terms, which indicated that the *p*-value of these terms was significant [25]. On the other hand, a greater F-value is more desirable, since lower values could produce more error in the model. As shown in Table 2, the F-value was 144.14 and 71.67 for R_1_ and R_2_, respectively, which implies that the model was significant. Another essential parameter in the data analysis was lack of fit, which must be a non-significant value in order to fit the model [24]. In the present study it was noted that lack of fit was 2.01 and 1.32, with related *p*-values of 0.3490 and 0.4580, for R_1_ and R_2_, respectively, indicating a good, non-significant lack of fit that was sufficient for the expected responses.

**Table 1:** Experimental design for various FA-loaded NEs, along with their detected values of response.

Formula	Independent Variables			Dependent Responses	
	X_1_ (g)	X_2_ (g)	X_3_ (g)	R_1_ (nm)	R_2_ (%)
NE 1	2.5	0.5	1.5	191 ± 2.7	45.5 ± 2.3
NE 2	2.5	1	1	215 ± 3.6	42.4 ± 2.4
NE 3	2	0.5	2	163 ± 2.6	58.0 ± 2.6
NE 4	2	1	1.5	159 ± 2.0	61.0 ± 3.1
NE 5	2	1.5	1	171 ± 3.1	53.3 ± 2.7
NE 6	1.5	0.5	1.5	144 ± 2.8	65.3 ± 3.9
NE 7	1.5	1	1	136 ± 2.4	68.1 ± 2.8
NE 8	1.5	1	2	124 ± 2.2	71.3 ± 3.3
NE 9	2	1	1.5	155 ± 2.6	62.3 ± 3.6
NE 10	2.5	1	2	210 ± 3.0	43.0 ± 2.9
NE 11	2.5	1.5	1.5	226 ± 3.3	40.1 ± 2.5
NE 12	1.5	1.5	1.5	116 ± 1.9	75.6 ± 4.5
NE 13	2	1.5	2	152 ± 2.2	57.4 ± 2.7
NE 14	2	0.5	1	168 ± 2.0	55.3 ± 2.9
NE 15	2	1	1.5	160 ± 2.5	59.4 ± 2.8

X_1_: oil concentration; X_2_: Tween 80 concentration; X_3_: Transcutol^®^ P concentration; R_1_: particle size; R_2_: in vitro release.

### 2.2. Characterization of FA-Loaded NE Formulations

#### 2.2.1. Influence of Independent Variables on Particle Size

Estimation of the particle size of the developed FA-loaded NE preparations was carried out, since it was regarded as an important factor for characterizing the formulation. As shown in Table 1, the particle size of all developed formulations ranged from 116 ± 1.9 to 226 ± 3.3, indicating that the particle size of the formulations was nanoscale. It was noteworthy that all of the independent variables exerted a measurable influence on the studied response R_1_. It was apparent that increasing the oil concentration (X_1_) from 1.5 to 2.5 g caused a relative increase in the particle size of all NE formulations, which could be attributable to an increase in the dispersed phase [26]. This result was similar to that obtained by Sakeena et al., who stated that the particle size of palm oil ester nanoemulsion was increased when increasing the oil concentration [27]. Conversely, an inverse relationship was detected between NE particle size (R_1_) and Tween 80 concentration (X_2_), since it was observed that increasing X_2_ while using the same oil concentration (X_1_) was accompanied with decreasing Y_1_. In fact, the small particle size of NE was characterized by a large surface area, requiring an increase in X_2_ for coating it [28]. This result was consistent with the findings of Chuacharoen et al., who reported that a smaller particle size of curcumin nanoemulsion was detected at higher concentrations of lecithin, which acts as surfactant [29]. Moreover, higher X_2_ resulted in a larger oil–water interface, which could lower R_1_ [30]. With regard to Transcutol^®^ P (X_3_) behaving like a co-surfactant, it was noted that when keeping X_1_ and X_2_ constant, increasing X_3_ would decrease the NE particle size. Additionally, combining X_1_ and X_2_ and increasing their concentrations would provide a further decrease in NE particle size. This impact could be attributed to that surfactant and co-surfactant causing a reduction in the interfacial tension, which would subsequently reduce the particle size [31]. 

Our aforementioned observations could be emphasized by a mathematical equation generated by BBD, and illustrated the influence of the various independent variables used (X_1_, X_2_, and X_3_) on the dependent response R_1_. As is known, the positive sign identifies the synergistic action, while the negative one describes the antagonistic effect [32]. We noticed a considerable positive impact of the X_1_ variable on the response (R_1_); however, X_2_ and X_3_ exerted a negative influence.

R_1_ = 158 + 40.25 X_1_ − 0.125 X_2_ − 5.125 X_3_ + 15.75 X_1_X_2_ + 1.75 X_1_X_3_ − 3.5 X_2_X_3_ + 9.5 X_1_^2^ + 1.75 X_2_^2^ + 3.75 X_3_^2^

**Figure 1:**
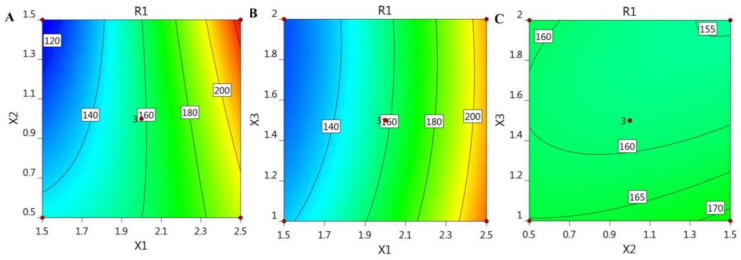
2D contour graphs demonstrating the influence of the independent factors (**A**) X_1_ and X_2_, (**B**) X_1_ and X_3_, and (**C**) X_2_ and X_3_ on particle size responses (R_1_).

**Figure 2:**
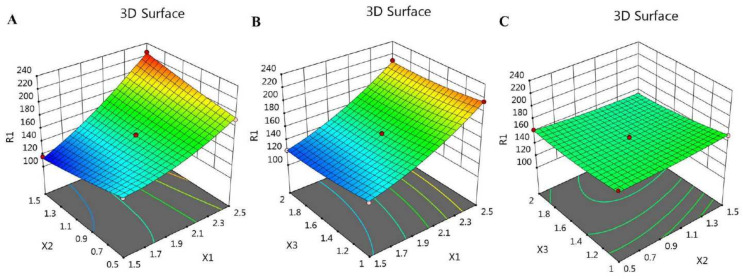
3D response surface plots demonstrating the influence of the independent factors (**A**) X_1_ and X_2_, (**B**) X_1_ and X_3_, and (**C**) X_2_ and X_3_ on particle size responses (R_1_).

In addition, the BBD software plotted certain graphs, as exhibited in Figure 1 and Figure 2, which show 2D contour graphs and 3D response surface plots, respectively, that explain the influence of the different independent variables on the R_1_ response.

**Figure 3:**
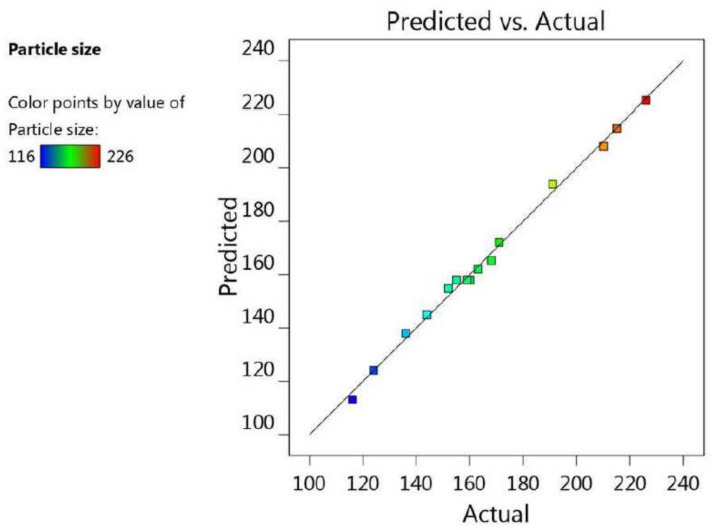
Predicted versus actual plot representing the linear correlation between values for particle size response (R_1_).

Additionally, the fit of the statistics and the linearity of the data were cleared up using the adjusted R^2^ value (0.9892) and the predicted R^2^ value (0.9517). It was noticeable that the difference between them was less than 0.2, and both were very close to 1, which is a requisite for fitting the model; therefore, both of them were in reasonable agreement with one another, as shown in Table 2 and Figure 3. Furthermore, the R^2^ value was 0.9962, indicating good support of the system to the model, in addition to the adequate precision value (40.8966) measuring the signal-to-noise ratio, which indicates an adequate signal, and proves that the model could be used to navigate the design space.

#### 2.2.2. Influence of Independent Variables on In Vitro Release Study (R_2_)

**Figure 4:**
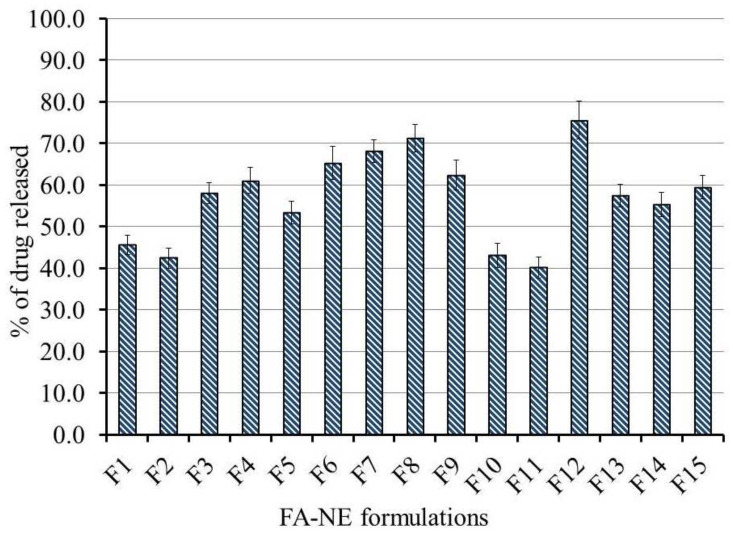
In vitro release of FA from various NE formulations kept at 32 °C using pH 5.5 phosphate buffer for 6 h. Results are presented as the mean values of three determinations ± SD.

Assessment of in vitro release of FA from the fabricated NE formulations was effectively performed, and the profile of the study is depicted in Figure 4. In line with the data in Table 1, the percentage of FA released from all NE formulations varied from 40.1 ± 2.5 to 75.6 ± 4.5%. It was apparent that the independent variables exerted a considerable effect on the in vitro release pattern. Accordingly, increasing X_1_ resulted in a subsequent decrease in R_2_, which could be attributed to the greater particle size due to higher oil concentration, which provides a smaller surface area and, subsequently, decreases the % of drug released [33]. Conversely, upon increasing X_2_ and X_3_, a substantial increase in the percentage of FA release (R_2_) was detected. This could be explained by the previous report by Eid et al., who stated that Tween 80 possessed the greatest solubilizing ability for FA, and led to improved drug release [34]. Moreover, the presence of a surfactant and co-surfactant in the formulation helps in forming NEs with small particle size via lowering the surface tension, which increases the in vitro drug release [35]. Predominantly, the particle size of the formulation represents a key factor in the process of drug release, since maximum drug release is achieved by systems with small particle size [36].

The previously identified influence of the independent variables X_1_, X_2_, and X_3_ on the in vitro release response (R_2_) could be further illustrated by mathematical modeling represented by the equation below. It was clear that X_1_ had a negative impact on the R_2_ response; however, X_2_ and X_3_ displayed a positive synergistic effect.

R_2_ = 60.9 − 13.6625 X_1_ + 0.2875 X_2_ + 1.325 X_3_ − 3.925 X_1_X_2_ − 0.65 X_1_X_3_ + 0.35 X_2_X_3_ − 2.0375 X_1_^2^ − 2.2375 X_2_^2^ − 2.6625 X_3_^2^

**Figure 5:**
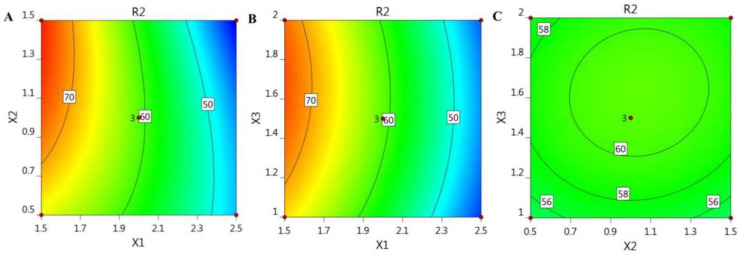
2D contour graphs signifying the influence of the independent factors (**A**) X_1_ and X_2_, (**B**) X_1_ and X_3_, and (**C**) X_2_ and X_3_ on in vitro release response (R_2_).

**Figure 6:**
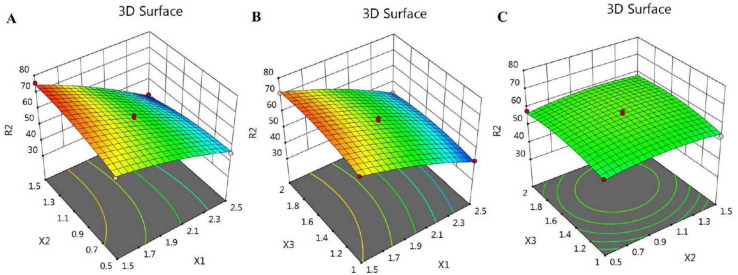
3D response surface plots signifying the influence of the independent factors (**A**) X_1_ and X_2_, (**B**) X_1_ and X_3_, and (**C**) X_2_ and X_3_ on in vitro release response (R_2_).

For illustrative representation of the relationships between the three independent variables and the studied response (R_2_), certain model graphs were created by the software, as exemplified in Figure 5, which shows 2D contour graphs, and Figure 6, showing 3D surface plots.

**Figure 7:**
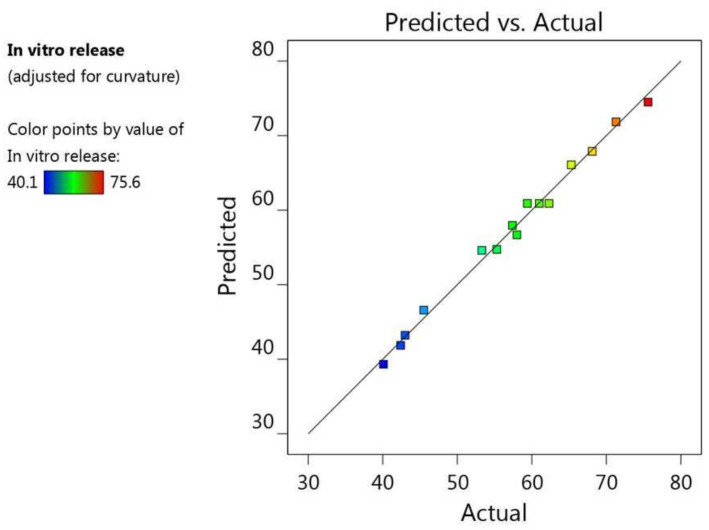
Predicted versus actual plot representing the linear correlation between values for in vitro release response (R_2_).

Figure 7, along with the data in Table 2, shows the linear interrelation between the predicted and actual values of response. It was observed that the difference between the predicted and adjusted R^2^ was less than 0.2, and both were close to 1, since their values were 0.9124 and 0.9785, respectively; thus, they were in credible compliance, and were highly correlated with one another. Likewise, the R^2^ was 0.9923, which implies good precision and accuracy of the experimental values. The settled adequate precision value (27.1516) was greater than 4, denoting the fitting of the model, and suggesting that the model could navigate the design space.

### 2.3. Optimization and Verification of the Examined Variables

**Figure 8:**
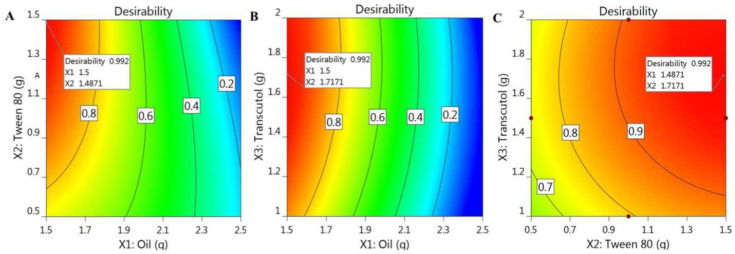
Optimization figures screening the influence of (**A**) X_1_ and X_2_, (**B**) X_1_ and X_3_, and (**C**) X_3_ and X_2_ on overall desirability.

To attain the best NE formulation possessing the optimal features and suitable levels of constraints, the observed responses were optimized by applying a numerical optimization technique. This technique was carried out with BBD software using certain assigned criteria that were predicted to provide the optimized formula. The assigned criteria in the present investigation were to minimize particle size and to maximize the % of in vitro drug release. Taking into consideration the data offered in the point prediction post-analysis sector, the desirability function was employed with a value between 0 and 1, revealing to what extent the response data were close to the target value [37]. The anticipated actual independent variable values were 1.5 g of myrrh essential oil, 1.48 g of Tween 80, and 1.717 g of Transcutol^®^ P. Consistent with Figure 8, which shows the higher desirability value (0.992), the predicted values of the responses were 109.6 nm for R_1_ and 75% for R_2_. With reference to the predicted results, the NE formulation that was considered to be the optimized formula was fabricated and evaluated for its responses. It was readily apparent that the attained values were compatible with the predicted ones, as displayed in Table 3.

Based on the optimization process, the optimized FA-NE was incorporated successfully into a Na CMC hydrogel base, providing a fine and stable FA-NEG formulation that exhibited no sign of phase separation at room temperature or in the refrigerator

### 2.4. Characterization

#### 2.4.1. Visual Inspection

The fabricated FA-NEG was examined visually for its physical properties and compared with its relative FA-G, as listed in Table 4. The formulations were white, creamy, and viscous, with a homogeneous and smooth appearance. 

#### 2.4.2. Measuring pH Value

The pH value is a very important factor for topical formulations, as it determines whether or not the formulation could result in irritation. Values of pH for all formulations were 6.39 ± 0.27 and 6.61 ± 0.23 for FA-G and FA-NEG, respectively. It was clear that the values were within an acceptable range, making them safe to apply topically, avoiding the risk of skin irritation. This is consistent with the results of Razzaq et al., where the pH of the fabricated nanoemulgel ranged between 6.16 and 6.65 [38].

#### 2.4.3. Viscosity

Regarding the formulations’ rheological behavior, it was necessary to estimate, since the viscosity of the preparation affects the diffusion rate of the drug and controls the in vitro release [39]. As shown in Table 4, the viscosity of the FA formulations was 15,245.0 ± 360.3 and 25,265.0 ± 400.2 for FA-G and FA-NEG, respectively, which seemed to be within the proper range for topical application. Presumably, there was a significant difference between the viscosity of FA-G and of FA-NEG (*p* < 0.05); however, both formulations seemed to be suitable for skin application. Our result was consistent with those of Bolla et al., since the viscosity of the developed ibuprofen emulgel preparations was in the range of 29.659 cP [40]. 

#### 2.4.4. Spreadability

The spreadability of the semisolid formulations is considered to be an important parameter, as it helps in determining what amount of the formulation to spread uniformly when applied on the skin. Spreadability was measured for the formulations under investigation, and was recorded as 40.5 ± 2.5 mm and 33.6 ± 2.3 mm for FA-G and FA-NEG, respectively, which proved to be excellent despite the significant difference that was detected between the two formulations (*p* < 0.05). Our result was consistent with those of Soliman et al., where the spreadability of a curcumin nanoemulgel was about 53.5 mm [7].

**Table 4:** Characterization of optimized FA-G and FA-NEG.

Characteristics	FA-G	FA-NEG
Visual Inspection	White, smooth, and homogeneous	White, creamy, smooth, and homogeneous
pH	6.39 ± 0.27	6.61 ± 0.23
Viscosity (cP)	15,245.0 ± 360.3	25,265.0 ± 400.2 *
Spreadability (mm)	40.5 ± 2.5	33.6 ± 2.3 *

Results are presented as the mean values of three determinations ± SD, using Student’s *t*-test; * *p* < 0.05 compared to FA-G.

### 2.5. In Vitro Release Study of FA from Developed Formulations

**Figure 9:**
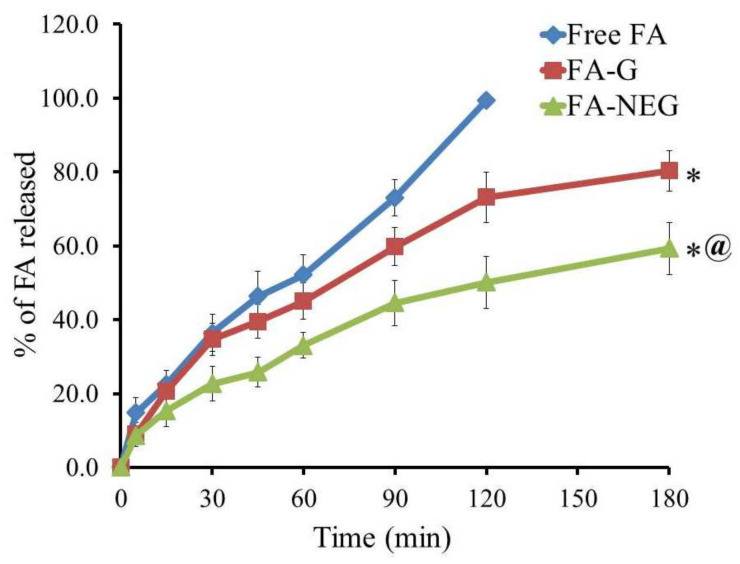
Outline of in vitro release of FA from FA-G and FA-NEG compared to FA as a free drug, using pH 5.5 phosphate buffer at 32 ± 0.5 °C. Results are expressed as the mean ± SD of three trials; * *p* < 0.05 compared to free FA; @ *p* < 0.05 compared to the FA-G formulation.

In vitro release of FA through a cellophane membrane from the developed FA-G and FA-NEG was measured and compared to FA free powder, and the outline of the results is portrayed in Figure 9. After 60 min, almost 52.2% of FA was dissolved in the medium, reaching 99.5% after 120 min. On the other hand, the percentage of FA released from FA-G and FA-NEG was 80.3 ± 5.53 and 59.3 ± 5.1%, respectively, over 180 min. It was apparent that the percentage of FA released from the formulations under investigation was significantly different compared to that released from free FA (*p* < 0.05). Furthermore, the percentage of FA released from FA-G seemed to be significantly greater than that released from FA-NEG (*p* < 0.05). This observation was related to the higher aqueous content in the gel preparation, which facilitated the drug release into the vehicle. Contrariwise, the lower percentage of FA released from the NEG formulation was definitely attributed to the higher viscosity of the formulation, owing to the incorporation of myrrh essential oil, which worked to slow down the diffusion of the drug [41]. Additionally, the NEG formulation behaved like a drug reservoir, where the drug to be released would pass through the inner phase to the outer one, which might have slowed the release rate [42]. The obtained results confirm that different dosage forms with different excipients can strongly influence the in vitro release of the drug from the formulation [43]. 

### 2.6. Kinetic Study

**Figure 10:**
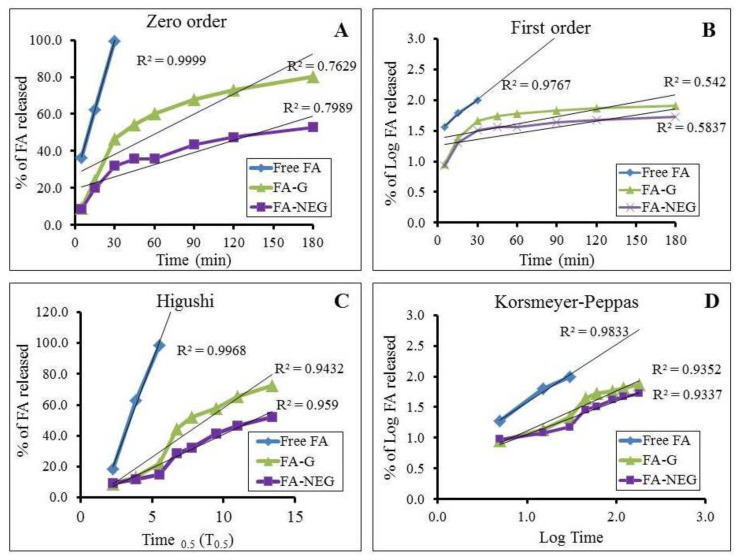
Percentage of drug released from developed FA formulations against free FA, and their kinetic analysis, according to (**A**) zero-order (**B**) first-order, (**C**) Higuchi, and (**D**) Korsmeyer–Peppas models.

In order to distinguish the mechanisms through which the drug is released from the formulation across the membrane, a kinetic study was implemented for all formulations under investigation, and the results are displayed in Table 5 and Figure 10. The amount of drug released versus time curve was constructed, and the greatest value of r^2^ and the most linear plot were determined. It was revealed that the release of FA from all formulations followed Higuchi kinetics, confirming that the drug was released from a matrix type with perfect sink conditions [44]. Higuchi modeling is always used to describe the drug dissolution from transdermal systems [45].

### 2.7. Stability Study

**Figure 11:**
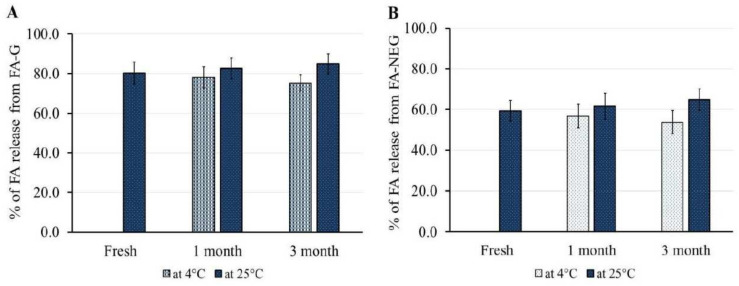
Outline of stability studies for (**A**) FA-G and (**B**) FA-NEG formulations for 1 and 3 months at 4 °C and 25 °C in terms of in vitro drug release, compared to their corresponding freshly prepared formulations. Data are expressed as means ± SD for three experiments.

The stability of the formulated FA-G and FA-NEG was investigated upon storage at 4 ± 1 °C and 25 ± 1 °C, and the study protocol was performed for 1 and 3 months. As per the data shown in Table 6, it was established that FA-G and FA-NEG showed non-significant variation in their physical properties under both storage conditions after 1 and 3 months when compared with their corresponding fresh preparations (*p* < 0.05). However, a significant difference was detected between FA-G and FA-NEG in terms of the assessed viscosity and spreadability (*p* < 0.05). Despite this significant variation, the physical properties of both formulations were within an adequate range to facilitate their topical application. Furthermore, Figure 11 shows that there was no significant difference in FA release from FA-G and FA-NEG throughout the storage period under both conditions, compared to their respective freshly prepared formulations (*p* < 0.05). The former results confirm the formulations’ stability, and emphasize the potency of nanoemulgels as nanocarriers. 

### 2.8. Ex Vivo Permeation Study

**Figure 12:**
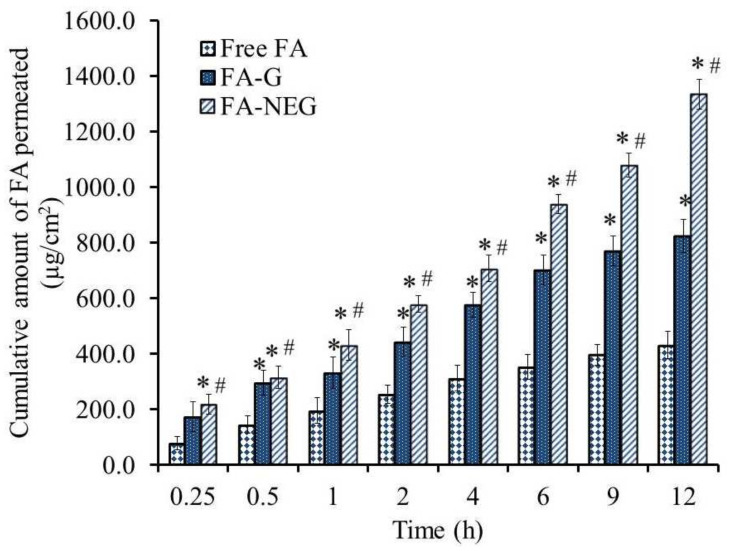
Ex vivo permeation study profile of FA from diverse preparations (free FA, FA-G, and FA-NEG) across a rat skin membrane. Results are expressed as means ± SD (*n* = 3); * *p* < 0.05 compared to free FA; # *p* < 0.05 compared to the FA-G formulation.

The most efficient way of assessing the effectiveness of active pharmaceutical ingredients via topical application is to carry out skin permeability studies using excised animal skin and evaluate certain permeability parameters [46]. Accordingly, the skin permeability of FA from selected formulations across animal skin was evaluated. Table 7 and Figure 12 show the permeation parameters and the permeability of FA from the investigated formulations. The results could be arranged in the following order: FA-NEG > FA-G > free FA. A significantly lower amount of FA permeated from the free FA suspension compared to the other formulations under study, exhibiting an SSTF value of 35.9 ± 4.1 µg/cm^2^·h (*p* < 0.05). Conversely, the flux of FA from FA-NEG was significantly enhanced by 3.10 ± 0.13-fold, showing an SSTF of 111.2 ± 4.5 µg/cm^2^·h compared to that from FA-G (68.7 ± 5.0 µg/cm^2^·h), displaying an ER value 1.91 ± 0.14 (*p* < 0.05). Primarily, integrating Transcutol^®^ P as a penetration enhancer in FA-G and FA-NEG formulations enhanced the drug permeation through rat skin. It was previously reported by Osborne et al. that Transcutol^®^ P could promptly improve the penetration through the stratum corneum which, in turn, would modify the drug penetration [47]. On the other hand, the higher flux of FA from FA-NEG than from the FA-G formulation could be ascribed to the presence of a surfactant with colloidal characteristics, in addition to myrrh oil, which could act as a permeation enhancer, helping to improve the permeability [48]. Furthermore, the higher permeation of FA from FA-NEG could be due to its composition, since it was composed of a nanoemulsion that could possibly diffuse through the membrane’s narrow pores [49]. It was stated in a previous study that nanoemulgels could feasibly enhance the permeation into deep skin layers compared to other formulations [42]. 

### 2.9. In Vivo Study

#### Skin Irritation Test

Careful examination of animal back skin treated with the investigated formulations was performed for checking any sensitivity reactions that might occur. No inflammation, irritation, erythema, or edema was recognized on the inspected area throughout the whole 7 days of the investigation, reflecting the safety of the formulations.

### 2.10. Antibacterial Study

**Figure 13:**
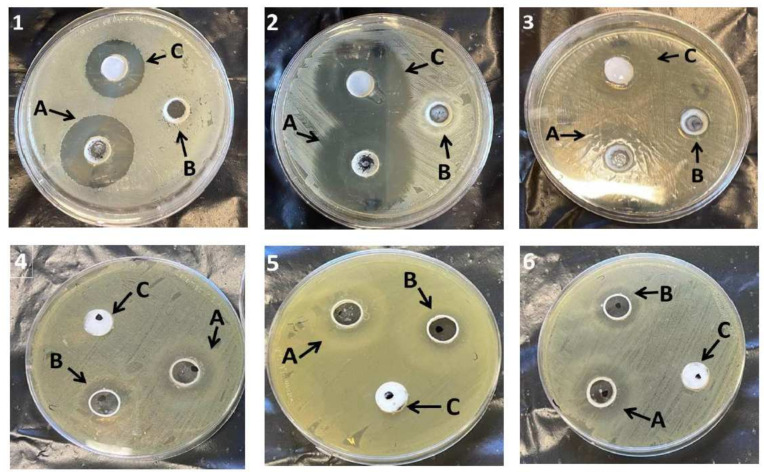
Inhibition zone diameters caused by various formulations—(**A**) FA-NEG, (**B**) placebo NEG, and (**C**) marketed FA—on different organisms: (**1**) *Bacillus subtilis*, (**2**) *Staphylococcus aureus*, (**3**) *Enterococcus faecalis*, (**4**) *Candida albicans*, (**5**) *Shigella*, and (**6**) *Escherichia coli*.

The antibacterial activity of the NEG formulations with and without FA was assessed against the number of organisms by estimating the inhibition zone, and was compared to the marketed cream, as displayed in Table 8 and Figure 13. It was apparent that FA-NEG was active against *Staphylococcus aureus*, *Bacillus subtilis*, and *Enterococcus faecalis*, and showed a significantly greater zone of inhibition compared to that resulting from placebo NEG and the marketed formulation (*p* < 0.05). Additionally, FA-NEG and placebo NEG exhibited a significant inhibition zone against *Candida albicans*, *Shigella*, and *E. coli* compared to the marketed formulation, which showed a negative effect against these bacteria (*p* < 0.05). Our results were well matched with previous findings that showed negative and very low antibacterial effects of marketed FA against *E. coli* and *Shigella* compared to fusidic acid nanoemulgels [34]. Moreover, Aksu et al. demonstrated similar findings, since in situ fusidic acid gel showed higher antibacterial activity than the marketed fusidic acid product [2]. Interestingly, NEG free from FA showed slight inhibition of the bacteria, which could be attributed to the incorporation of myrrh oil, which possesses antibacterial activity. Moreover, the higher antibacterial activity of FA-NEG could be ascribed to the antibacterial synergism of FA and myrrh oil. 

## 3. Conclusions

Fusidic acid was incorporated into various nanoemulsions prepared with myrrh essential oil, which were fabricated and optimized using the BBD approach. The optimized nanoemulsion was successfully incorporated into a hydrogel base, providing FA-NEG for topical application. The developed FA-NEG exhibited acceptable physical properties to be applied topically. It showed enhanced skin permeation and no irritation following skin application. FA-NEG and the blank nanoemulgel showed great antibacterial activity when compared with marketed fusidic acid. The investigation revealed the great influence of myrrh essential oil and fusidic acid as antibacterial agents, and highlighted the synergistic action between them. In conclusion, nanoemulgel systems incorporating fusidic acid and myrrh essential oil could be promising nanocarriers for providing antibacterial effects via topical delivery. Our future goal is to explore the influence of the formulation’s activity on animal wounds infected with different types of bacteria, comparing the healing rates with those provided by marketed fusidic acid products.

## 4. Materials and Methods

### 4.1. Materials

Fusidic acid was obtained from Saudi Pharmaceutical Industries & Medical Appliances Corporation (SPIMACO ADDWAEIH, KSA, Riyadh, Saudi Arabia). Diethylene glycol monoethyl ether (Transcutol^®^ P) was purchased from Gattefosse SAS (Cedex, Saint-Priest, France). Non-ionic surfactants (polysorbate 80; Tween 80) and the gelling agent sodium carboxymethyl cellulose (Na CMC) were purchased from Sigma-Aldrich Co. (St. Louis, MO, USA). Myrrh essential oil was purchased from NOW^®^ Essential Oils (NOW Foods, Bloomingdale, IL, USA). All other solvents and chemicals were of analytical grade.

### 4.2. QbD Approach Using BBD

A matrix of 15 formulae was constructed using BBD, which is one of the most tolerable designs for scrutinizing the data and obtaining the optimized formula. Accordingly, a three-factor, three-level (33) factorial design was built using Design-Expert version 12.0 software (Stat-Ease, Minneapolis, MN, USA). As per Table 9, the independent variables that were investigated represented the oil concentration, surfactant concentration, and co-surfactant concentration, signified as A, B, and C, respectively. Each of these independent factors was assigned at three levels (−1, 0, or 1), demonstrating the lowest, central, and highest values, respectively. The impact of the previous factors A, B, and C was considered on behalf of two responses, namely, particle size (R_1_) and in vitro release (R_2_). Data obtained were analyzed statistically using analysis of variance (ANOVA), in addition to definite model graphs that were plotted using the software and mathematical polynomial equations, which could suggest a clarification for the obtained response, as follows:

R = bo + b1A + b2B + b3C + b12AB + b13AC + b23BC + b11A2 + b22B2 + b33C2(1)

where R characterizes the detected response, b0 is the intercept; (b1–b3), (b12–b23), and (b11–b33) are the regression coefficients, A, B, and C indicate the main factors, AB, AC, and BC specify the interactions between the main factors, and A2, B2, and C2 signify the polynomial terms.

### 4.3. Development of FA-Loaded NE

Several NE formulations loaded with FA were developed according to the method previously reported by Elsewedy et al., using the quantified amounts of ingredients as presented in Table 1. Primarily, 500 mg of FA was dissolved in various concentrations of myrrh essential oil and Transcutol^®^ P, and subjected to vortexing using a classic advanced vortex mixer (VELP Scientifica, Usmate, Italy) to provide the oily phase. Afterwards, different concentrations of surfactant (Tween 80) were added to distilled water, representing the aqueous phase, and were vortexed well. The aqueous phase was gradually added to the oily phase, homogenized using a high-shear homogenizer (T 25 digital Ultra-Turrax, IKA, Staufen, Germany), and kept for 15 min at 20,000 rpm. Following the homogenization process, the emulsion developed instantly, and was exposed to 30 s of sonication using a probe sonicator (XL-2000, Qsonica, Newtown, CT, USA) until homogeneous NE was obtained [50].

### 4.4. Characterization of FA-Loaded NE Formulations

#### 4.4.1. Particle Determination

Analysis of the particle size of FA-loaded NEs was accomplished at 25 °C using a Zetasizer apparatus (Malvern Instruments Ltd., Worcestershire, UK), by estimating the dynamic light scattering at a scattering angle of 90°.

#### 4.4.2. In Vitro Release Study from NE Formulations

In order to determine the percentage of FA released from the prepared NE formulations, the present study was conducted using the ERWEKA dissolution system (ERWEKA, GmbH, Heusenstamm, Germany). Concisely, a specified amount of each NE formulation was placed on a plate fixed to glass tubes and covered with a cellophane membrane (MWCO 2000–15,000) from one side, while the other side of the tubes was attached to the apparatus. The tubes were submerged in 500 mL of pH 5.5 phosphate buffer, which represented the release medium. The temperature was maintained at 32 ± 0.5 °C, and the apparatus was allowed to rotate at 50 rpm. Samples of about 2 mL was withdrawn from each examined formulation at predetermined time intervals and up to 6 h. The withdrawn sample was replaced with an equal amount of the pH 5.5 vehicle phosphate buffer. The absorbance was checked spectrophotometrically using a UV spectrophotometer (JENWAY 6305, Bibby Scientific Ltd., Staffs, UK) at λmax 285 nm. The investigation was carried out using three vessels per formulation (*n* = 3). 

### 4.5. Development of FA Loaded into a Myrrh-Oil-Based Nanoemulgel (FA-NEG)

Although applying NE formulations over the skin would provide better penetration through the skin and enhanced values for permeation parameters, it is a well-known fact that more viscous formulations are more appropriate for topical applications, since they can be spread evenly and are not easily detached from the area of treatment [51]. Therefore, it was necessary to incorporate the optimized FA-NE into one of the innovative topical formulations—namely, NEG. Additionally, to authenticate the role and the therapeutic influence of myrrh essential oil, an FA-based hydrogel (FA-G) was developed. 

#### 4.5.1. Developing FA-G

At the beginning, 25 g of a Na CMC hydrogel base was prepared by scattering 500 mg of the gelling agent in distilled water. The mixture was blended until a homogeneous gel base was obtained, using a magnetic stirrer (JeioTech TM-14SB, Medline Scientific, Oxfordshire, UK). Next, 500 mg of FA was dissolved in a specific amount of Transcutol^®^ P, and then mixed well for 5 min and added to the pre-formulated hydrogel base [52].

#### 4.5.2. Developing FA-NEG

For developing FA-NEG, it was essential to fabricate and mix both FA-NE and the gel base. FA-NE was fabricated by preparing and mixing the oily phase with the aqueous phase, as reported in Section 2.3. The optimized FA-NE was mixed with the Na CMC hydrogel base previously prepared using 500 mg of gelling agent sprinkled over distilled water. Both were mixed well for 5 min using a mixer (Heidolph RZR 1, Heidolph Instruments, Schwabach, Germany) until fine FA-NEG was formulated [53]. 

### 4.6. Characterization

#### 4.6.1. Visual Inspection

The developed FA-G and FA-NEG were visually examined for their physical properties, including appearance, color, and homogeneity. 

#### 4.6.2. Measuring pH Value

The pH of the developed formulations was evaluated because of its prominence in certifying the safety of the topical formulation to be applied without causing skin irritation. This measurement was assessed at room temperature using a standardized pH meter (MW802, Milwaukee Instruments, Szeged, Hungary) [12]. 

#### 4.6.3. Viscosity

The viscosity of the developed topical formulations was estimated at 25 ± 0.3 °C utilizing a Brookfield viscometer (DV-II + Pro, Middleboro, MA, USA), with spindle R5 rotated at 0.5 rpm [50].

#### 4.6.4. Spreadability

A spreadability technique was employed for detecting the capability of the formulation to spread readily when applied to the treated area, which is a critical parameter for transdermal preparations. Two glass slides (25 cm × 25 cm) were used, and 0.5 g of each formulation was placed between them. A specific load (500 g) was placed over the slides for 1 min, and then the diameter of the spreading area was measured to determine the spreadability value [54].

### 4.7. In Vitro Release Study of FA from the Developed Topical Formulations

The percentage of FA released from FA-G and FA-NEG was estimated using the ERWEKA dissolution system (ERWEKA, GmbH, Heusenstamm, Germany), and was compared to that released from the FA solution. The investigation was performed following the same procedure described in Section 4.4.2.

### 4.8. Kinetic Study

The outline of the in vitro release study from all formulations was plotted using various kinetic modes, in order to detect the correlation coefficient (r^2^) and the release kinetics. The highest value of (r^2^) for the drug release data, in addition to a linear plot, would be related to the best fit model [55]. A graph plotting drug concentrations against time (T) was produced by employing the following models:

a. A zero-order kinetic model that shows the percentage of drug released against T.
b. A first-order kinetic that shows the Log percentage of drug remaining against T.
c. Higuchi’s model that shows the percentage of drug released against the square root of T.
d. A Korsmeyer–Peppas model that shows the Log percentage of drug released against log T.

### 4.9. Stability Study

Consistent with the guidelines of the International Conference on Harmonization (ICH), stability studies of FA-G and FA-NEG were conducted. The study was performed for 1 and 3 months under 2 different temperature conditions—4 ± 1 °C and 25 ± 1 °C—with humidity of 60%, estimating specific parameters such as physical properties and in vitro release [56].

### 4.10. Animal

Animals were provided by the breeding center at the College of Science, King Faisal University. Male Wistar rats weighing 220 to 250 g were kept under optimal laboratory conditions, with free access to food and water, and at ambient temperature (25 ± 2 °C). All experimental protocols related to animals were in accordance with the Ethical Conduct for Use of Animals, and were approved by the Research Ethics Committee (REC) at King Faisal University (KFU-REC-2021-Oct—EA00080). 

### 4.11. Ex Vivo Study

#### 4.11.1. Preparing Animal Skin

Dorsal hair of male Wistar rats was removed with care using an electric clipper, followed by killing the animals using ketamine overdose [57]. The skin was separated from the animal and the adipose tissue was removed. The separated skin was maintained for further study by hydrating it in pH 5.5 phosphate buffer, and was kept at 4 °C. 

#### 4.11.2. Ex Vivo Permeation Study

The modified Franz diffusion cell assembled in our lab was operated to detect the permeability of FA through animal skin from the developed formulations [55,57]. The diffusion medium was composed of 100 mL of pH 5.5 phosphate buffer kept at 32 ± 0.5 °C. The skin was fixed well to a glass tube in the apparatus, where the upper epidermis was facing the formulation, while the dermis was facing the release medium. The system was covered with a parafilm (Bemis, Oshkosh, WI, USA) to prevent medium evaporation. The apparatus was allowed to be stirred continuously at 100 rpm. Then, 1 mL of the sample was withdrawn and replaced with an equal volume of the fresh medium over a period of 12 h. In order to compare the drug transfer with the permeation properties between the investigated formulations, certain constraints related to the permeation process across the skin were evaluated. These parameters included steady-state transdermal flux (SSTF), which denotes the amount of permeated drug/(area × time), and the enhancement ratio (ER), which indicates the SSTF of test/SSTF of control. Each experiment was conducted in triplicate, and the results were presented as mean values ± SD [54].

### 4.12. In Vivo Study

#### Skin Irritation Test

Irritation testing is an essential examination for the safety evaluation of the formulation; consequently, male Wistar rats were used for performing such investigation. Rats were prepared before proceeding into the study by shaving the hair of their back using a digital clipper. The inspected formulations were evenly applied to the shaved area of the rat, followed by observing the skin for 7 days to be assessed and scored for any reaction. The skin reaction was scored as 0, 1, 2, or 3, indicating no reaction, slight, moderate, and severe erythema with or without edema, respectively [36].

### 4.13. Antibacterial Study

In order to evaluate the antibacterial activity of the formulated FA-NEG, various organisms obtained from the American Type Culture Collection (ATCC) were used, namely, *Staphylococcus aureus* (ATCC 29213), *Bacillus subtilis* (ATCC 10400), *Enterococcus faecalis* (ATCC 773), *Candida albicans* (ATCC 90028), *Shigella* (ATCC 11060), and *Escherichia coli* (*E. coli*) (ATCC 25922). The culture medium was prepared using Mueller–Hinton agar poured into a sterile Petri dish. Three wells each of 6 mm diameter were made and packed with FA-NEG, NEG without drug (placebo NEG), and the marketed FA cream. The plates were incubated for 24 h at 37 ± 1 °C and then evaluated for their antibacterial performance by measuring the inhibition zone diameter. Experiments were carried out in triplicate.

### 4.14. Statistical Analysis

All data were displayed as the mean ± standard deviation (SD). For multiple groups, comparisons were performed using one-way analysis of variance (ANOVA), followed by the least significant difference (LSD) as a post hoc test. The data were considered significance if *p* < 0.05. All statistical analysis was carried out using SPSS statistics software, version 14 (IBM Corporation, Armonk, NY, USA).

## Figures and Tables

**Table 2:** Results of statistical analysis of dependent variables.

Source	R_1_		R_2_	
	F-Value	*p*-Value	F-Value	*p*-Value
Model	144.14	<0.0001 *	71.67	<0.0001 *
X_1_	1152.04	<0.0001 *	593.17	<0.0001 *
X_2_	0.0111	0.9201	0.2627	0.6301
X_3_	18.68	0.0076 *	5.58	0.0646 *
X_1_X_2_	88.20	0.0002 *	24.48	0.0043 *
X_2_X_3_	1.09	0.3445	0.6713	0.4499
X_3_X_1_	4.36	0.0912	0.1946	0.6775
X_1_^2^	29.62	0.0028 *	6.09	0.0567
X_2_^2^	1.01	0.3621	7.34	0.0423 *
X_3_^2^	4.62	0.0844	10.40	0.0233 *
Lack of Fit	2.01	0.3490	1.32	0.4580
R^2^ analysis				
R²	0.9962		0.9923	
Adjusted R²	0.9892		0.9785	
Predicted R^2^	0.9517		0.9124	
Adequate Precision	40.8966		27.1516	
Model				
Remark	Quadratic		Quadratic	

X_1_: oil concentration (mg); X_2_: Tween 80 concentration (mg); X_3_: Transcutol^®^ P; R_1_: particle size (nm); R_2_: in vitro release (%); *: significant.

**Table 3:** Predicted and observed values of response at the optimal conditions.

Dependent Response	Predicted Values	Observed Values
R_1_ (nm)	109.667 ± 3.35	113.6 ± 3.21
R_2_ (%)	75.0 ± 1.58	71.9 ± 2.65

**Table 5:** Kinetics of FA release from the studied formulations.

Formulation	Zero-Order Kinetic (r^2^)	First-Order Kinetic (r^2^)	Higuchi Kinetic (r^2^)	Korsmeyer–Peppas Kinetic (r^2^)
FA Suspension	0.937	0.811	0.974	0.934
FA-G	0.756	0.521	0.893	0.861
FA-NEG	0.864	0.667	0.967	0.964

**Table 6:** Physical characterization of FA-G and FA-NEG after 1 and 3 months of storage at a relative humidity 60% and temperatures of 4 °C and 25 °C.

Properties	Temperature	FA-G	FA-NEG	FA-G	FA-NEG
		1 Month		3 Months	
Physical Inspection	4 °C	No phase separation	No phase separation	No phase separation	No phase separation
	25 °C				
pH	4 °C	6.51 ± 0.29	6.70 ± 0.19	6.58 ±0.30	6.68 ± 0.20
	25 °C	6.41 ± 0.35	6.55 ± 0.20	6.54 ± 0.29	6.72 ± 0.27
Viscosity (cP)	4 °C	16,150 ± 736	26,090 ± 641 *	16,720 ± 687	27,050 ± 589 *
	25 °C	14,575 ± 566	24,510 ± 720 *	14,050 ± 655	24,510 ± 720 *
Spreadability (mm)	4 °C	39.3 ± 2.7	32.4 ± 2.5 *	38.5 ± 2.6	31.7 ± 2.5 *
	25 °C	41.2 ± 2.4	34.5 ± 1.9 *	40.3 ± 2.4	34.5 ± 1.9 *

Values are expressed as means ± SD; * *p* < 0.05 compared to FA-G.

**Table 7:** Skin permeation parameters of the investigated formulations.

Formula	SSTF µg/cm^2^·h	ER
Free FA	35.9 ± 4.1	1
FA-G	68.7 ± 5.1 *	1.91 ± 0.14 *
FA-NEG	111.2 ± 4.5 * #	3.10 ± 0.13 * #

Values are expressed as means ± SD; * *p* < 0.05 compared to free FA; # *p* < 0.05 compared to the FA-G formulation.

**Table 8:** Microbiological activity of the tested formulations against various organisms.

Bacterial Type	Inhibition Zone (cm)		
	FA-NEG	Placebo NEG	FA Cream
*Bacillus subtilis*	3.6 ± 0.18	3.4 ± 0.19	2.8 ± 0.21
*Staphylococcus aureus*	4.4 ± 0.17	2.2 ± 0.10	3.9 ± 0.15
*Enterococcus faecalis*	3.1 ± 0.15	0.9 ± 0.08	2.5 ± 0.10
*Candida albicans*	2.2 ± 0.12	2.0 ± 0.14	Negative
*Shigella*	2.8 ± 0.16	2.7 ± 0.15	Negative
*Escherichia coli*	2.3 ± 0.10	1.7 ± 0.18	Negative

**Table 9:** BBD data presenting the independent variables with their levels of variation and the examined dependent responses.

Independent Variable	Symbol	Level of Variation		
		Lowest (−1)	Central (0)	Highest (1)
Oil Concentration (g)	A	1.5	2.0	2.5
Tween 80 (g)	B	0.5	1.0	1.5
Transcutol^®^ P (g)	C	1.0	1.5	2.0
**Dependent Variable**	**Symbol**	**Constraints**		
Particle Size (nm)	R_1_	Minimize		
In vitro release (%)	R_2_	Maximize		

## Data Availability

Not applicable.
